# Diagnostic Value of Ultrasound in Detection of Biliary Tract Complications After Liver Transplantation

**DOI:** 10.5812/hepatmon.6003

**Published:** 2013-01-20

**Authors:** Andrej Potthoff, Anreas Hahn, Stefan Kubicka, Andrea Schneider, Jochen Wedemeyer, Juergen Klempnauer, Michael Manns, Michael Gebel, Bita Boozari

**Affiliations:** 1 Department of Gastroenterology, Hannover Medical School, Hannover, Germany; 2 Department of Biometrics, Hannover Medical School, Hannover, Germany; 3 Department of Internal Medicine, Klinikum Robert Koch Gehrden, Gehrden, Germany; 4 Department of Visceral and Transplant Surgery, Hannover Medical School, Hannover, Germany

**Keywords:** Complications, Liver transplantation, Cholangiography

## Abstract

**Background:**

Biliary complications are significant source of morbidity after liver transplantation (LT). Cholangiography is the gold standard for diagnosis and specification of biliary complications.

**Objectives:**

Detailed analyses of ultrasound (US) as a safe imaging method in this regard are still lacking. Therefore we analyzed systematically the diagnostic value of US in these patients.

**Patients and Methods:**

Retrospectively, 128 liver graft recipients and their clinical data were analyzed. All patients had a standardized US examination. The findings of US were compared to cholangiographic results in 42 patients. Following statistical analyses were performed: descriptive statistics, sensitivity, specificity, positive and negative predictive values (PPV, NPV).

**Results:**

42 patients had 54 different biliary complications (Anastomotic stenosis (AS) n = 33, ischemic type biliary lesions (ITBL) n = 18 and leakage n = 3). US detected n = 22/42 (52%) patients with biliary complications. The sensitivity, specificity, PPV and NPV of US were: 61%, 100%, 100%, 79% (95CI, 36-86%) for ITBL and 24%, 100, 100%, 31% (95CI, 9-46 %) for AS, respectively.

**Conclusions:**

US examination had no false positive rate. Therefore, it may be helpful as a first screening modality. But for the direct diagnosis of the biliary complication US is not sensitive enough.

## 1. Background

Biliary complications occur in 9-35 % of liver transplant recipients (1-8). It is reported as an uncommon cause of mortality, but represent a significant source of morbidity (9). Many authors differentiate clinically between the early and late biliary complications. Early complications such as leaks and strictures have often technical causes and occur predominantly within the first three months after LT. Late complications are more likely to be complex and have multiple causes (9). Cholangiography methods such as endoscopic retrograde cholangio-pancreatography (ERCP) and percutaneous transhepatic cholangiodrainage (PTCD) are the diagnostic and interventional standards in this regard. The ERC (P) (Endoscopic retrograde cholangio (pancreatography)) is the method of choice in patients with duct-to-duct reconstruction. PTCD is usually preferred for patients with a biliodigestive anastomosis if balloon enteroscopic approach fails (9). Magnetic resonance cholangiopancreatography (MRCP) is reported as a reliable diagnostic tool in detection and exclusion of biliary complications (10). The low spatial resolution and interference due to superposition of extra hepatic fluid are limitations of this method (10). Further limitations are high costs, claustrophobia and contrast agent associated complications such as Nephrogenic Systemic Fibrosis. Ultrasound (US) is the first diagnostic tool in many centers. There are only few published studies regarding to the diagnostic value of US in detection of biliary tract complications after LT. In older studies, remarkable ultrasound findings were reported to be predictive for the cholangiographic diagnosis of biliary obstruction or the generalized ductal changes with a specificity of 98 % and 100 % respectively (11). The sensitivity of US in direct detection of biliary complications is reported, in a single publication so far, as very low (12).

## 2. Objectives

The aim of this study was therefore to investigate systematically the diagnostic value of ultrasound in direct detection of biliary complications in liver recipients.

## 3. Patients and Methods

The study protocol was designed according to the declaration of Helsinki 2008. Written informed consent was obtained from all included patients. For this study we analyzed retrospectively, based on the hospital electronic data bank, all liver recipients between January first 2005 through December 31th 2006 recruited primarily. A total of 189 adult patients received liver grafts through this time period. The inclusion criterions for this studywas an ultrasound examination after liver transplantation independent from the time point or reason of the ultrasound examination. Therefore we excluded 49 patients without documented ultrasound examination after LT Then we looked for those patients who had a cholangiography. The indication for cholangiography was provided by the patient history and clinical data independent from their ultrasound results. We compared the results of cholangiography as the gold standard method with ultrasound findings. The exclusion criterion was a time interval longer than four weeks between the ultrasound examination and the cholangiography. Therefore 12 more patients were excluded. The final analysis was therefore performed with the remaining 128 patients, from them 42 had both US and cholangiography. Due to the clinical course of our department, all ultrasound examinations were performed before the cholangiography examination. Therefore, the sonographers were blinded to results of the cholangiography but not to the clinical results. All patients were examined by gastroenterologists with DEGUM (German association of ultrasound in medicine) level II to III ultrasound training using ultrasound equipment Aplio (Toshiba, Japan), Sonoline Antares (Siemens, Germany) and Elegra Sonoline Advanced (Siemens, Germany) with convex arrays C 3-6 MHz (Toshiba, Japan), CH4-1 and three, five C40H (Siemens, Germany), respectively. Systematic B-mode examinations of all abdominal organs including retro peritoneum were performed routinely. The examinations mode for all organs were performed following the recommendations of the German Association of Ultrasound in Medicine (DEGUM). Color Doppler and duplex measurements of the right hepatic artery and the portal vein were performed in an oblique intercostal array position and during mid-inspiration with the patient’s right arm abducted. Maximum velocity of the portal vein [(P) Vmax (Maximum velocity of the portal vein)], peak systolic velocity of the hepatic artery [(A) Vmax (Maximum systolic velocity)], end diastolic velocity of the hepatic artery [(A) Vmin (Maximum end diastolic velocity)] and resistive index of the hepatic artery RI (Resistance index) were measured in front and behind the anastomosis in each case. Settings such as gain filter and pulse-repetition frequencies were adjusted as needed for optimal signal detection to prevent artifacts. Types of biliary complications and pathologies According to the biliary complications literature were defined as following: Anastomotic stenosis (AS), ischemic type biliary lesion (ITBL) and leakage (9). The definition of biliary complications on cholangiography is reported as following (13):

### 3.1. AS on Cholangiography

Stricture with narrowing of the biliary lumen of common bile duct CBD (Common bile duct) at the level of the anastomosis.

### 3.2. ITBL on Cholangiography

Single or multiple strictures of the biliary tree in liver transplant recipients. Type I: extra hepatic lesion, Type II: intrahepatic lesion, Type III: extra- and intrahepatic lesion.

### 3.3. Leakage on Cholangiography

Extravasations of contrast agent after injection into the biliary tree. The definition for biliary complications on ERCP followed the advice of published studies (13-15). The definition of biliary complications on US has been stated only partially (16). We defined them as follows:

### 3.4. AS on US

**Figure 1:**
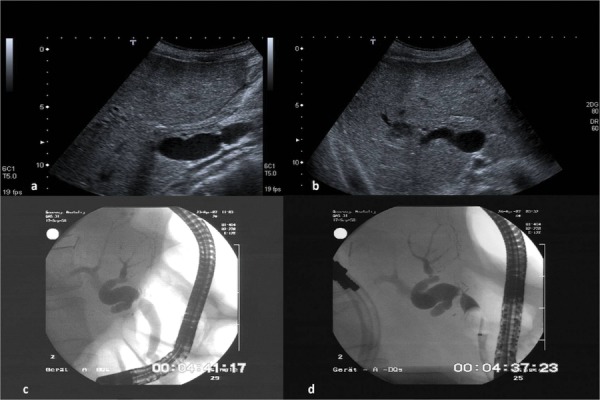
The Images Show a Stenosis at The Level of The Anastomosis With Intrahepatic Dilatation of The Bile Ducts Figure 1a demonstrates a liver recipient with a biliodigestive anastomosis in a longitudinal position of the ultrasound probe in the media clavicular line. Dilatated gut loop with fluid in liver hilum. Figure 1b demonstrates the right lobe of the same patient in a sub costal view. Dilatated right common bile duct with a peripheral abscess (arrow) due to the stenosis of the bile duct at the level of the bilio digestive anastomosis. Figure 1c and 1d demonstrate the corresponding ERCP images without and with blocked balloon catheter

Bile duct dilatation intra (> 3mm) and / or extra hepatic (> 10mm) on either anastomosis sides of CBD (Figure 1a  and b).

### 3.5. ITBL on US

**Figure 2:**
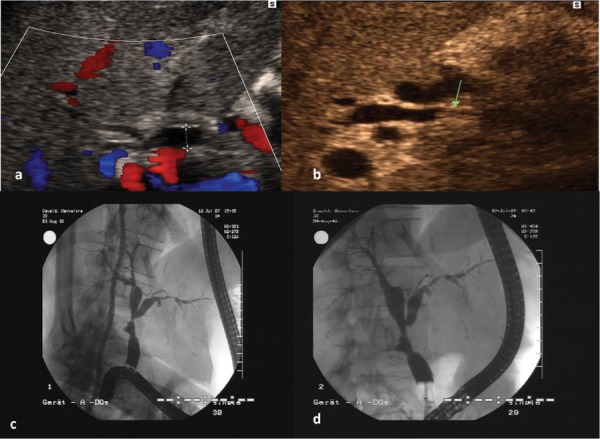
The Images Show Ischemic Type Biliary Lesions (ITBL) Signs at the Level of The Anastomosis as Well as The Intrahepatic Bile Ducts Figure 2a demonstrates a centrally dilated right common bile duct of a liver recipient in color mode. Figure 2b demonstrates the same patient with partially thickening of the wall (arrow) of the common right bile duct representing an ischemic biliary type lesion. Figure 2c and 2d demonstrate the corresponding ERCP images without and with blocked balloon catheter

Bile ducts with thickened wall and / or presence of material in lumen, with or without consecutive dilatation (Figure 2a and b).

### 3.6. Leakage on US

Liquid formation in liver hilum and or sub capsular region with ascites. Confirmation of bilirubin in fluid collection after US-guided fluid aspiration. In this study we differentiated between biliary complications and general biliary pathologies. Biliary pathologies on US were defined as: Cholangitis, intrahepatic dilatation, extra hepatic dilatation, change of the wall of the bile ducts, sludge, stone, abscess, stent or aerobilia in the bile ducts.

### 3.7. Statistical Analysis

The statistical evaluation was performed using the statistical package for social sciences for Windows^™^ (SPSS). Descriptive statistics were performed. Mean values and standard deviations (mean ± SD) of parametric data were analyzed and compared using T-test. Correlations were performed using Pearson correlation test (r = correlations factor). Finally, sensitivity, specificity, positive predictive value PPV (Positive predictive value) and negative predictive value NPV (Negative predictive value) were calculated using cross tabulations. The analysis was performed on a patient by patient basis.

## 4. Results

### 4.1. Descriptive Statistics

128 patients (54 % males, mean age 50 years) were included in the analysis; Main indications for LT (Liver transplantation) were viral hepatitis 24 %, alcoholic liver disease 19 % and PSC (Primary Sclerosing Cholangitis) (18 %). clinical data of the patients are shown in Table 1. The ultrasound findings are demonstrated in Table 2. Table 3 demonstrates the mean value of laboratory data in the group of patients with and without biliary complications. The mean value of ALT (Alanin aminotransferase), AST (Aspartate aminotransferase), ALP (Alkaline phosphatase), GGT (Gamma glytamyl transferase) and bilirubin were significantly higher in the group of patients with biliary complications. A total of 42 Patients have been received a comparable US and cholangiography. The US was able to detect a biliary complication in 22 (52.4 %) of them. The US was also helpful in detection of gross general biliary pathologies (Table 4).

**Table 1:** Clinical Data of Patients (N) After Orthotropic Liver Transplantation

	Patients	Mean ± SD	Median
**Gender, No. (%)**			
Female	59 (46.1)	-	-
Male	69 (53.9)	-	-
**Age, y, range**	24-70	50.22 ± 11.1	52
**Body mass index, kg/m^2^, range**	16.7-35.6	25.2 ± 4.4	24.5
**Split liver, No. (%)**	18 (14.1)		
**Graft size, kg, range**	0.7-2.9	1.6 ± 0.4	1.6
**Duct to duct anastomosis**	103 (80.5)		
**Biliodigestive anastomosis**	25 (19.5)		
**Stenting therapy, No. (%)**	24 (18.8)	12 ± 8.6	10.5
**Duration of stenting therapy, mo, range**	0-38	12 ± 8.6	10.5
**Death**	17 (13.3)	17 (13.3)	
**Biliary Complications on Cholangiography (ERC/PTC)**			
Within 3 months	20 (47.6)		
Within 1 year	17 (40.5)		
After 1 year	5 (11.9)		
**Other imaging modalities**			
CT	16 (38.1)		
MRCP	3 (7.1)		
CT and MRCP	9 (21.4)		
**Liver disease**			
Chronic viral hepatitis	31 (24.2)		
Alcoholic	24 (18.8)		
Primary sclerosing cholangi	23 (18)		
Others a	20 (15.6)		
Autoimmune b	12 (9.4)		
Unknown	8 (6.3)		
Cystic	6 (4.7)		
Acute liver failure	4 (3.1)		
Additional tumor c	29 (22.7)		

Abbreviations: CT, computed topography, MRCP, Magnetic resonance Cholangio Pancreatography

^a^Others include: Hepatocellular carcinoma, oxalises, cystic fibrosis, secondary sclerosing cholangitis, Amyloidosis, carcinoid disease, alpha1 antitrypsin deficiency, glycogenesis, non-alcoholic steatohepatitis, glycogenesis, familiar hypercholesterolemia, Budd chiari syndrome

^b^Autoimmune includes: Autoimmune hepatitis and primary biliary cirrhosis

^c^n = 3 cholangiocellular carcinoma, n = 26 hepatocellular carcinomas

**Table 2:** Ultrasound (US)

	Patients	Mean ± SD	Median
**Open artery **a	125 (97.6)		
**Resistive index, range b**	0.4 – 0.85		
**Stenosis of arterial anastomosis**	8 (6.3)		
**Open PV c**	126 (98.4)		
**Portal vein velocity, cm/s d, range**	9 – 184	0.67 ± 0.1	0.68
**Stenosis of PV anastomosis**	13 (10.2)		
**Open HV e**	122 (95.3)		
**Thrombosis of HV**	2 (1.6)	31.3 ± 20.8	26
**Lymph nodes**	14 (10.9)		
**Ascites**	29 (22.7)		
**Changed Liver parenchyma**	26 (20.3)		
**Splenomegaly f**	68 (45.3)		
**Total**	128 (100)		

^a^n = 3 missing values.

^b^103 (80.5%) of patients were in resistive index.

^c^PV, Portal Vein, n = 2 Thrombosis of portal vein.

^d^Portal vein velocity measured in 119 patients (92.9%).

^e^HV, Hepatic Vein, n = 2 Thrombosis of the liver vein, n=4 missing values.

^f^n= 8 missing values, n = 3 with status post splenectomy.

**Table 3:** Mean Value of Laboratory Data in Patients With and Without Biliary Complications

	Normal Value, Range	With Complication	Without Complication	P value
**CRP, mg/l**	8	19.9 ± 40.8	20.1 ± 30.9	None Significant
**ALT, U/l**		152.9 ± 155.1	61.7 ± 134.5	0.002
Female	Up to 34			
Male	Up to 45			
**AST, U/l**		132.1 ± 166.6	51.8 ± 124.5	0.008
Female	Up to 31			
Male	Up to 35			
**ALP, U/l**		458.8 ± 443	142.7 ± 192.4	0.0001
Female	35 – 104			
Male	40 – 129			
**GGT, U/l**		502.8 ± 403.1	128.3 ± 386.7	0.0001
Female	Up to 38			
Male	Up to 55			
**GLDH, U/l**		28 ± 42.8	14 ± 70.1	None Significant
Female	Up to 5			
Male	Up to 7			
**Bilirubin, μmol/l**	17	61.1 ± 65.5	21.4 ± 40.9	0.001
**Total, No.**		42	80	

Abbreviations: CRP: C-Reactive Protein , GLDH: Glutamat Dehydrogenase; ALP, Alkaline phosphatase; ALT, Alanin aminotransferase; GGT, Gamma glytamyl transferase; AST, Aspartate aminotransferase.

**Table 4:** US Versus Cholangiography in Detection of General Biliary Pathologies (n = 128)

	ERC/PTC, N (%)	USN (%)
**Only on ERCP**	7 (5.5)	
**Only on US a**		13 (10.1)
**Cholangitis**		10 (7.8) b
**Cholangiocellular carcinoma**		1 (0.8)^c^
**Ischemic type biliary lesions**		1 (0.8) d
**Complex stenosis**		1 (0.8) e
**Intrahepatic dilatation**		30 (23.4)
**Extra hepatic dilatation**		23 (18)
**Change of the wall**		27 (21.1)
**On ERCP and US**		
Sludge	15 (11.1)	7 (5.5)
Stone	7 (5.5)	3 (2.3)
Abscess	4 (3.1)	3 (2.3)
Stent patency	9 (7)	8 (6.3)
Pneumobilia	10 (7.8)	9 (7)
**Total**	42 (100)	22 (52)

Abbreviations: ERCP, endoscopic retrograde cholangio-pancreatography; US, Ultrasound; ERC/PTC, endoscopic retrograde cholangio/percutaneous transhepatic cholangio-drainage.

^a^11 of 13 patients had no cholangiography procedure.

^b^The accuracy of the diagnosis was proved by other imaging modalities as well as biochemical tests.

^c^These patients had no ERC or PTC.

^d^This case was missed by ERC.

^e^This case was not detected by PTC due to interventional difficulties.

### 4.2. Sensitivity, Specificity, PPV and NPV

Table 5 demonstrates the comparison between US and cholangiography in detection of biliary complications in liver recipients. The sensitivity, specificity, PPV and NPV of US for detection of ITBL were 61 %, 100 %, 100 % and 79 % (95CI (Confidence Interval), 36-86 %), respectively. The sensitivity, specificity, PPV and NPV of US for detection of AS were 24 %, 100, 100 % and 31 % (95CI, 9-46 %), respectively. The sensitivity, specificity, PPV and NPV of US for detection of leakage were 67 %, 98 %, 67 % and 98 % (95CI, 9-99 %) respectively.

**Table 5:** Comparison of Ultrasound in Detection of Biliary Complications With Cholangiography

	Total, No.	AS, No.	ITBL, No.	Leakage, No.
**ERC/PTC**	42	33	18	3
**Correct pos. on US**		8/17	11/18	2/3
**Correct neg. on US**		9/9	24/35	38
**False pos. on US**		0	0	1/2
**False neg. on US**		25/33	7/18	1/2

Abbreviations: AS: Anastomotic Stenosis, ITBL: Ischemic Type Biliary Lesion; ERC/PTC, endoscopic retrograde cholangio/percutaneous transhepatic cholangio- drainage.

### 4.3. Discrepancy Between US and Cholangiography

**Figure 3:**
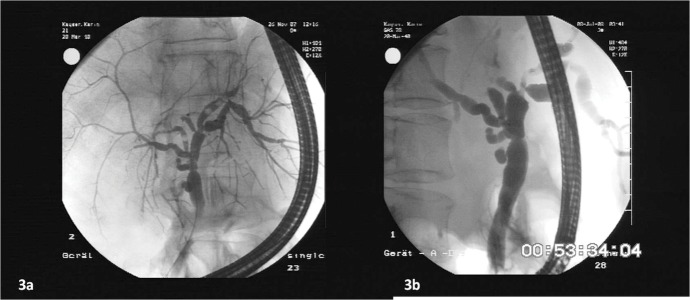
Figure 3a and Figure 3b Demonstrate a Diffuse ITBL Without Bile Duct Dilatations on ERCP. The Diagnosis Was Missed Completely by Ultrasound

**Figure 4:**
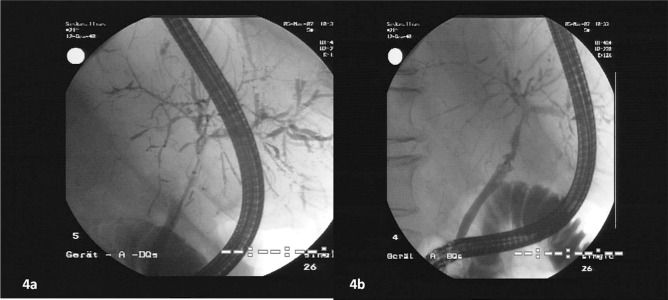
Figure 4a Demonstrates ERCP Images of a Patient With ITBL Who was Diagnosed Only on Ultrasound. ERCP Missed the Diagnosis at This Time. Figure 4b is The Same Patient Seven Months Later. By The Second Examination the Diagnosis ITBL Was Also Confirmed by ERCP.

In two cases (4,7 %), the biliary complication was detected only on US: In one case the diagnosis of diffuse ITBL was missed by ERCP, while US could detect the thickening of the small bile ducts without dilatations; the definite diagnosis of ITBL was then confirmed with ERCP later and histologically after re-transplantation (Figure 3a and b). In the second case with status post hepaticojejunostomy, PTC (Percutaneous transhepatic cholangiography) could not be performed due to absence of intra hepatic biliary dilatation. The diagnosis of ITBL was also proven by histology after surgical resection and porto-jejunostomy. These two cases are not considered in the analysis because the gold standard was cholangiography. On the other hand, from 22 patients with biliary complications on US 7 (16, 6 %) patients were diagnosed only on cholangiography and were missed completely by US: five cases with AS and two cases with ITBL. In 3 of 5 patients with AS no intrahepatic dilatation of the bile ducts was seen on ultrasound (Figure 4a and b). The corresponding ERCP diagnosed discreet and rough stenosis in two cases and mild stenosis in one case (no bile duct narrowing on ERCP, difficulty of passage from the anastomosis with the blocked balloon or delayed outflow of contrast medium above the level of the anastomosis). In the other two cases the examiner did not notice the caliber difference of CBD at the level of the anastomosis. From the two missed ITBL cases, in one patient the ITBL was developed only in the left biliary system and in the second case a diffuse ITBL without dominant dilatation was present. Usually all patients with biliary complications receive routine follow up every three months in our department. All seven patients who were primarily missed by US had detectable sonographic signs of biliary complications after three months. In 15 from 22 patients with biliary complications on US, this method was able to detect at least one complication which led to further investigations and verification of other complications.

### 4.4. Correlation Between Vascular and Biliary Complications

A total of 19 cases showed remarkable vascular changes on US: 11 in the portal or venous anastomoses and eight in the arterial anastomoses. We could not show any correlations between the frequency of biliary complications and RI (r = 0.025, P = 0.8) or vascular complications such as portal vein thrombosis/stenosis (r = 0.03, P = 0.7) and thrombosis/stenosis of the arterial anastomosis (r = 0.095, P = 0.3). From eight patients with arterial complications, six (75 %) developed biliary complications. All six patients developed ITBL and two cases had additionally an anastomotic stenosis. Patients with selective complications of the portal anastomosis showed no biliary complications (n = 11).

### 4.5. Additional Biliary Pathologies Apart From Biliary Complications Seen by US

Apart from biliary complications, in 11 cases (8.6 %) ultrasound could detect further biliary pathologies without the necessity of cholangiography intervention. In 10 patients ultrasound suggested a cholangitis. The diagnosis was confirmed with the clinical picture, biochemical, other imaging modalities and follow up. In one case with PSC as underlying disease, US were the only method which could diagnose CCC (Cholangiocellular carcinoma) recurrence after LT through US + FNA. MRCP (Magnetic resonance cholangiopancreatography) and CT (Computed tomography) missed the diagnosis.

## 5. Discussion

US are the considered method of first choice for detection of biliary obstructions with consecutive dilatation of the bile duct since 20 years ago (17-19). Older studies have reported general sensitivity of 54 % for US in the detection of biliary pathologies after LT (20). Despite the fact some newer publications could prove sensitivity around 80 % (21, 22). Published data regarding the sensitivity of US in direct detection of biliary complications are very rare. Zoepf et al. reported a sensitivity of 68.4 % and 58.8 % for US in detection of biliary dilatation as indirect sign of AS and ITBL, respectively (12). After comparison of US, CT and MRI (Magnetic resonance imaging) for direct specification of the complications, the calculated sensitivities decrease to 0 %, 10 % and 22 %, respectively and was very low for all modalities (12). A direct comparison of our data to the study mentioned is not possible because Zoepf et al. considered only bile duct dilatation- which is an indirect sign of a biliary complication- as the only sonographic evidence for a biliary complication. For direct diagnosis of biliary complications in this study, the US examination was not sensitive enough. 14.1 % from all liver recipients and 42.9 % of all patients with biliary complications on cholangiography developed ITBL after LT. The sensitivity of US in detection of ITBL was 61 %. On the other hand in 4.8 % of the cases the diagnosis of ITBL was delayed by cholangiography and could be predicted by US only. In this study US had an impact of 66 % in detection of ITBL in the affected patients. Again 25.8 % of the transplanted population of this study developed an AS, which was the most frequent biliary complication (78.9 %) in our analysis as reported by other authors (23). For detection of AS, ultrasound had a very low sensitivity of 24 %. One explanation for the low sensitivity of US in detection of anastomosis stenosis might be the lack of systematic examination in some patients. In many of the missed cases in this study the CBD was examined only on one side. However, to detect stenosis of the anastomosis reliably, it is essential to examine the CBD on the donor and the recipient sides. The caliber difference of CBD pre and post anastomosis should always alert us to the diagnosis of AS. Slight and early stenosis on ERCP does not cause a dilatation of the bile ducts immediately. Due to the denervation of the implanted liver, it takes usually up to three months or more for development of a dilatation of the bile duct system that could be detected by US or other imaging modalities. This fact may be another explanation for limitations of US examination in this regard. The number of leakages and other rare complications in this study was too small for a reliable statistical analysis. Therefore an objective rating of US in this regard could not be performed.

**Figure 5:**
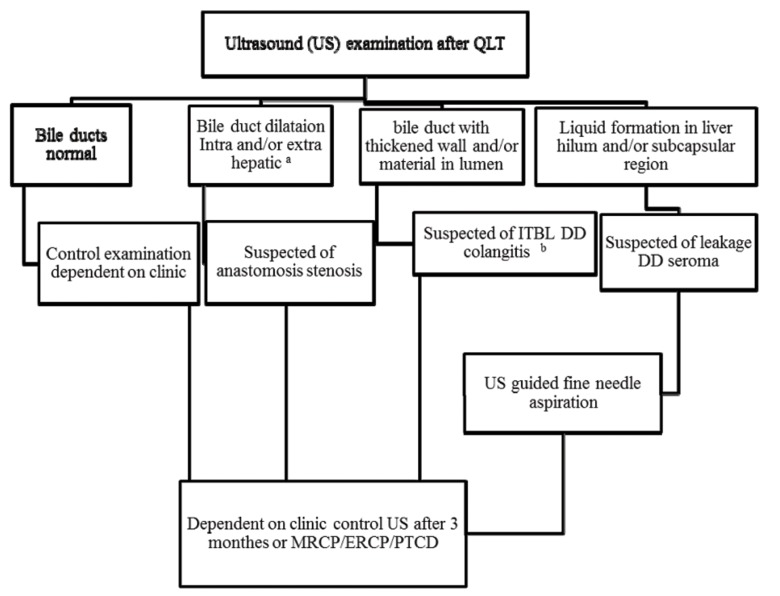
Screening Algorithm for Detection of Biliary Complications After Liver Transplantation a Segmental intra hepatic dilatations or common bile duct dilatations which is asymptomatic without any pathological biochemical tests should be controlled with US in follow up. b While the thickened wall of the bile ducts due to acute cholangitis disappears after antibiotic therapy it does not change in case of ischemic type biliary lesions (ITBL). This recommendation makes no claim to be complete and they do not account for rarities.

Limitation of this study is its retrospective character. Therefore the US and endoscopy diagnoses were not based on standardized protocols. We have tried to minimize this limitation by standardizing the findings of the imaging modalities retrospectively by clear definitions of the biliary pathologies. Based on the analyzed data we recommend the following screening algorithm for verification of biliary complications in liver recipients on US (Figure 5). This algorithm is only for orientation and does not consider all biliary pathologies. The data of this study show that US examination has a high specificity in detection of biliary pathologies. Therefore, it may be helpful as a first screening modality. But for direct diagnosis of the biliary complications it is not sensitive enough. In non-conclusive cases, if clinically possible, a repeating US examination after three months leads to a better verification of the pathologies of the biliary system. We presented an algorithm with low side effects and favorable cost profile for screening of liver recipients with biliary complications.
